# Differences in Fine-Root Biomass of Trees and Understory Vegetation among Stand Types in Subtropical Forests

**DOI:** 10.1371/journal.pone.0128894

**Published:** 2015-06-05

**Authors:** Xiaoli Fu, Jianlei Wang, Yuebao Di, Huimin Wang

**Affiliations:** Qianyanzhou Ecological Station, Key Laboratory of Ecosystem Network Observation and Modeling, Institute of Geographic Sciences and Natural Resources Research, Chinese Academy of Sciences, Beijing, 100101, China; University of Saskatchewan, CANADA

## Abstract

Variation of total fine-root biomass among types of tree stands has previously been attributed to the characteristics of the stand layers. The effects of the understory vegetation on total fine-root biomass are less well studied. We examined the variation of total fine-root biomass in subtropical tree stands at two sites of Datian and Huitong in China. The two sites have similar humid monsoon climate but different soil organic carbon. One examination compared two categories of basal areas (high vs. low basal area) in stands of single species. A second examination compared single-species and mixed stands with comparable basal areas. Low basal area did not correlate with low total fine-root biomass in the single-species stands. The increase in seedling density but decrease in stem density for the low basal area stands at Datian and the quite similar stand structures for the basal-area contrast at Huitong helped in the lack of association between basal area and total fine-root biomass at the two sites, respectively. The mixed stands also did not yield higher total fine-root biomasses. In addition to the lack of niche complementarity between tree species, the differences in stem and seedling densities and the belowground competition between the tree and non-tree species also contributed to the similarity of the total fine-root biomasses in the mixed and single-species stands. Across stand types, the more fertile site Datian yielded higher tree, non-tree and total fine-root biomasses than Huitong. However, the contribution of non-tree fine-root biomass to the total fine-root biomass was higher at Huitong (29.4%) than that at Datian (16.7%). This study suggests that the variation of total fine-root biomass across stand types not only was associated with the characteristics of trees, but also may be highly dependent on the understory layer.

## Introduction

Fine roots account for approximately 40% of the total net primary production in forest ecosystems globally and supply most of the nutrients and water to the aboveground biomass [1]. An increasing number of studies are examining the influence of tree species richness on fine-root biomass stock (FRB) at the stand level [2–6]. Belowground biodiversity/productivity studies of forest ecosystems have yielded three trends. The first is positive, whereby mixed forests with more tree species have more FRB than do corresponding single species stands [2–6], driven by niche complementarity (coexisting species occupy different soil space) and the “selection” effect (inclusion of a particularly productive species) [7]. Niche complementarity not only was observed in mature and closed forest stands but also occurred in very young tree communities before canopy closure [2, 3]. The second is negative, whereby mixed forests yield lower FRBs due to a very symmetrical interspecific competition among the tree species (the reciprocal interactions among species were equal in magnitude) [8–10]. The third is neutral, whereby the FRB is not affected by tree diversity, most likely due to a lack of complementarity in the vertical rooting patterns of the tree species present [11–12]. The effects of stem density and basal area on FRB are also controversial in single- species stands. For example, FRBs have been found to be lower [13], higher [14], or comparable [15, 16] in stands with lower stem densities compared to stands with higher stem densities. Stand age, canopy closure, and site fertility may influence the findings related to stem density, since considerable effects of these three factors on FRBs have been observed [17–20].

The influences of the understory vegetation in the belowground biodiversity/productivity and stem density studies, however, are less well studied. The FRB of understory could contribute substantially to the total FRB. For example, the FRB of the understory accounted for 20–70% of the total FRB in some temperate and boreal forests [21, 22]. Previous studies have shown that belowground competition with the shallow-rooted non-tree species negatively impacted the FRBs of trees [23]. In a subtropical savanna parkland, the depletion of soil resources induced by a shallow-rooted understory even led to the demise of the deeply rooted overstory plants [24]. The understory vegetation may be responsible for a significant variation in relative FRB of tree and non- tree species across different stand types.

The understory often forms a dense mat-like layer in forests in humid subtropical regions of the world [25]. The relative contribution of trees and the understory to the total FRB, however, has received very little attention [26]. We investigated the FRBs of tree and non-tree species for two categories of basal area (high vs. low) in stands of single tree species and in single-species vs. mixed stands with comparable basal areas in subtropical China. We conducted this study at two sites with similar climate but significantly different levels of soil organic carbon. The first aim of this study was to determine the influence of tree species and understory vegetation on the total FRB. The second aim of this study was to test how the FRBs and the association between stand type and FRB vary with study site.

## Materials and Methods

No specific permissions were required for these locations/activities. The specific locations of our study were in Datian County in Fujian Province, China (25°45′-25°46′N, 117°33′E) and in Huitong County in Hunan Province, China (26°52′N, 109°42′E). The field studies did not involve endangered or protected species.

### Study site

This study was conducted at two sites in China. One site was in Datian County in Fujian Province (25°45′-25°46′N, 117°33′E), at approximately 600 m a.s.l. The soil at this site is a red Humic Planosol (FAO classification) developed from an acidic sandy shale. The depth of the soil exceeds 1.0 m. The soil pH (0–10 cm depth) is 3.79. The other site was in Huitong County in Hunan Province (26°52′N, 109°42′E), at an altitude of 530–560 m a.s.l. The soil is a red Oxisol (FAO classification) derived predominantly from slate and shale [27]. The soil is 50–70 cm deep. The soil pH (0–10 cm depth) is 3.71. Soils at the two sites had similar soil total nitrogen but varied in soil organic carbon (S1 Table). The soil organic carbon (0–40 cm depth) at Datian is significantly higher than that at Huitong.

The two sites are approximately 1047 km apart but have the similar humid, mid-subtropical, monsoon climate. The mean annual temperatures are 15–18°C. The mean annual rainfall is approximately 1400 mm, and the rain mainly falls from March to August. Generally, precipitation decreased in July, to some degree, whereas the air temperature reached the maximum. The asynchronous seasonality between temperature and precipitation offered a possibility of frequent droughts in July. The zonal vegetation was evergreen broadleaved forest, which has nearly completely been removed by human activities. *Cunninghamia lanceolata* has become the major forest component. *C*. *lanceolata* stands are characterized by fast growing and high basal area [28]. For the stands of 15 m height, the basal area and volume can reach approximately 70 m^2^ ha ^-1^ and 540 m^3^ ha^-1^, respectively [29].

### Sampling design and data collection

We selected three stand types at each site. The three stand types were single-species *C*. *lanceolata* with high basal area (High), single-species *C*. *lanceolata* with low basal area (Low), and needle-broadleaf mixed forest (Mixed). At Datian, the three stand types were named as D-High, D-Low, and D-Mixed (mixed *C*. *lanceolata* and *Schima superba*). At Huitong, the three stand types were named as H-High, H-Low, and H-Mixed (mixed *C*. *lanceolata* and *Michelia macclurei*). We used High and Low to compare the FRBs in the two basal-area categories and used Low and Mixed to determine the influence of mixed stands on FRB based with similar basal-area [8, 11].

At Datian, D-Low and D-Mixed were established in 1991, and D-High was established in 1989. In 2013, the stands were in pre-mature stage. The stem-density ratio of *C*. *lanceolata* to *S*. *superba* in the mixed stand was approximately 3:1. The three stand types were similarly managed, such as weed-controlling during the first 3 years, thinning twice and fertilizing once during the first 10 years. The understory layers in all stands were dominated by dense ferns, with a small portion of dwarf shrubs. The dominant ferns were *Woodwardia japonica* (L. F.) Sm., *Dryopteris atrata* (Kunze) Ching, and *Dicranopteris dichotoma* (Thunb) Berhn. The shrubs were mainly *Ilex pubescens* Hook. et Am, *Clerodendrum cyrtophyllum* Turcz, *Loropetalum chinensis* (R. B R.) Oliv., *Fagus longipetiolata* Seem., and *Toxicodendron vernicifluum* (Stockes) F. A. Barkl. The soil organic carbon and total nitrogen contents to a depth of 40 cm were comparable for both comparisons (Table 1), helping to ensure comparable soil fertilities among the stand types. The non-tree aboveground biomasses were 214, 168, and 157 g m^-2^ in D-High, D-Low, and D-Mixed, respectively.

**Table 1:** Soil chemical properties (0–40 cm depth) of the stands (n = 5 plots per stand).

Parameter	Site	High	Low	Mixed	df	F	p-value
Sol total nitrogen (g kg^-1^)	Datian	0.94(±0.07)^a^	1.11(±0.09)^a^	1.10(±0.02)^a^	11	2.009	0.219
	Huitong	0.98(±0.04) ^a^	1.13(±0.04) ^a^	1.04(±0.03) ^a^	11	4.156	0.064
Soil organic carbon (g kg^-1^)	Datian	20.81(±1.81)^a^	22.98(±1.79)^a^	21.78(±1.41)^a^	11	0.417	0.647
	Huitong	15.53(±1.34) ^a^	15.23(±1.01) ^a^	16.08(±0.56)^a^	11	0.178	0.831

The stand types are single-species *C*. *lanceolata* with high basal area (High), single-species *C*. *lanceolata* with low basal area (Low), mixed *C*. *lanceolata* and *S*. *superba* / *M*. *macclurei* (Mixed). Same letters indicate not significant at *P* < 0.05.

At Huitong, H-High, H-Low, and H-Mixed, occupying a total of 10 ha, were established in the early spring of 1983. In 2013, the stands were in mature stage. The stem-density ratio of *C*. *lanceolata* to *M*. *macclurei* in the mixed stand was 4:1. The soil characteristics, including profile, texture, and mineral composition, were nearly identical among stand types in 1983 [27]. The soil organic-carbon and total-nitrogen contents to a depth of 40 cm were still comparable for both comparisons in 2013 (Table 1). Common management practices were used in the early stages of the three stand types, including weeding and chemical fertilization of the surface soil 1 m^2^ around the tree trunks [27]. The non-tree layers in the stands were dominated by dwarf shrubs, with a few herbs. The shrubs were mainly *Maesa japonica* (Thb.) Moritzi, *Kalopanax septemlobus* (Thunb.) Koidz, *C*. *cyrtophyllum*, and *Daphniphyllum macropodum Miq*. The herbs were mainly *Lophatherum gracile* Brongn., *Arthraxon hispidu*s (Thunb.) Makino, *Pteridium aquilinum* (L.) kuhn var. *latiusculum (Desv*.*) underw*., *Stenoloma chusanum* (Linn.) Ching, and *W*. *japonica*. The non-tree aboveground biomasses were 147, 154, and 145 g m^-2^ in H-High, H-Low, and H-Mixed, respectively.

The fine root production peaked in March and September while decreased substantially in January and July in subtropical regions [30]. The structures of the vegetation were surveyed and fine roots were sampled in May 2013 when the living FRBs of the non-tree species and *C*. *lanceolata* represented the annual average values and the ratio of living fine biomass to necromass was around 4:1 [30]. From January to May in 2013, the accumulated rainfall and average daily air temperature were 605 mm and 17.25°C at Datian while 688 mm and 12.92°C at Huitong. Datian had 14% lower accumulated rainfall but 34% higher average daily air temperature than Huitong. For each stand type, five 200-m^2^ circular plots were established, separated by more than 100 m. All plots were on the mid-slope with the slopes ranged from 25° to 30°. The diameter at breast-height (DBH) and species of trees with a DBH ≥2 cm were measured and recorded for the 30 plots at the two sites. The stand basal area by species was obtained at plot level. One representative 2 × 2 m subplot was delimited within each plot for the surveys of shrub layer. Within the subplot, all the shrub species were recorded and the tree species seedlings (mainly two-year old) were tallied. The herbaceous layer is relatively uniform. Therefore, one 1 × 1 m quadrat was delimited within the 2 × 2 m subplot to estimate the cover of herb and fern visually. Overall, 30 subplots and 30 quadrats were investigated for the shrub layer and herbaceous layer, respectively. The surveys were based on species identification, and no species overlapped the tree and understory layers. Shannon’s diversity index [31] of the non-tree species was calculated based on the method described in the previous study [32].

Within each circular plot (8 m radius), a random staring point was selected and nine soil cores were extracted at 1.5 m intervals along the centerline of the plot. The centerline passed through the understory survey subplot. To ensure the spatial heterogeneity was adequately sampled, the distance between sampling location and nearby tree stem ranged from 0.1 m to 1.5 m. Because an earlier study showed that the total FRB at 0–40 cm depth contributed 75% to the total FRB at 0–80 cm depth [33], soil cores (10 cm diameter) were collected at 0–10, 10–20, and 20–40 cm depths in the present study. The study yielded a total of 810 soil cores (9 locations × 3 depths × 5 plot replications × 3 stand types × 2 sites). We carefully extracted the living fine roots (diameter <2 mm) by hand. The pale-colored, elastic, and flexible roots free of decay and with a whitish cortex were considered as living roots [2]. The dead roots were not included in the later analysis. The living fine roots were washed and divided into the following species classes: *C*. *lanceolata* (brown exterior and red interior, coarsely structured with large root diameters), *S*. *superba* (white, more branched, finer structured with small root diameters), *M*. *macclurei* (milky white, coarser structured with smooth surfaces and large root diameters), and non-tree (whiter for the grasses and black and rigid for the ferns; the non-tree roots had fine hairs that the tree roots did not have). Finally, the sorted living fine roots within each soil core were dried at 70°C for 48 hours and weighed.

### Statistical analysis

We analyzed the two sites independently. We used a one-way analysis of variance and a multiple-comparison test (Tukey’s HSD test) to examine the effects of stand type on the structural traits of the stands and FRBs and to test the variation of FRBs among soil depths within stand types/sites and among stand types within soil depths. A two-factor analysis of variance within the General Linear Model procedure was carried out to explore whether the soil depth and stand type had any interaction effects on FRBs. We used independent sample *t*-tests to examine the differences of the chemical properties of the soil, structural traits of the stands, and FRBs between sites. These analyses were processed with SPSS Version 10.0 (SPSS Inc., Chicago, Illinois, USA).

## Results

### Stand structure

At Datian, the basal area, stem density, seedling density and shrub density varied with the stand types, however, the fern cover, herb cover, and Shannon’s diversity index of non-tree were similar among the three stand types (Table 2). For the comparison of basal area, D-High had higher stem density and shrub density but lower seedling density than D-Low. The difference in stem density for this comparison was around 80%. For the comparison of single-species and mixed stands, D-Mixed had a higher stem density but a lower seedling density than D-Low. D-Mixed had similar shrub density, compared with D-Low. The total cover of the fern and herb were 89.4%, 95% and 72.8% for D-High, D-Low, and D-Mixed, respectively.

**Table 2:** Structural traits (means±SE) of the stands (n = 5 plots per stand) at Datian.

Parameter	High	Low	Mixed	df	F	p-value
Basal area (m^2^ ha^-1^)						
*C*. *lanceolata*	69.75(±4.16)^a^	50.12(±3.57)^b^	42.43(±2.02)^b^	14	17.482	<0.001
*S*. *superba*	-	-	11.97(±1.49)			
Total	69.75^a^	50.12^b^	54.40(±2.96)^b^	14	8.242	0.006
Stem density (trees ha^-1^)						
*C*. *lanceolata*	2510(±128.84)^a^	1400(±93.54) ^c^	2010(± 76.49)^b^	14	29.715	<0.001
*S*. *superba*	-	-	710(± 29.15)			
Total	2510^a^	1400^b^	2720(± 84.56)^a^	14	46.440	<0.001
Seedling density (stems ha^-1^)						
*C*. *lanceolata*	5000(± 1581)^b^	30000(± 8329)^a^	4500(±500)^b^	14	8.842	0.004
*S*. *superba*	-	-	3000(±1118)			
Total	5000^b^	30000^a^	7500(±1581)^b^	14	7.647	0.007
Shrub density (stems ha^-1^)	14000(±2449)^a^	5000(±2622)^b^	8500(±1000)^ab^	14	4.450	0.036
Fern cover (total %)	80.8 (±5.3)^a^	82.4(±4.0)^a^	70.2(±8.3)^a^	14	1.178	0.341
Herb cover (total %)	8.6(±4.0)^a^	12.6(±2.3)^a^	2.6(±2.6)^a^	14	2.756	0.068
Shannon’s diversity index of non-tree	1.37(±0.16)^a^	1.33(±0.15)^a^	1.07(±0.14)^a^	14	1.591	0.244

The stand types are single-species *C*. *lanceolata* with high basal area (High), single-species *C*. *lanceolata* with low basal area (Low), and mixed *C*. *lanceolata* and *S*. *superba* (Mixed). Different letters indicate significant differences.

At Huitong, stand types showed no influence on the stand structure traits with the exception of seedling density (Table 3). No *C*. *lanceolata* seedling was observed in the three stand types. In H-Mixed, the *M*. *macclurei* seedling density was 3600 stems ha^-1^.

**Table 3:** Structural traits (means±SE) of the stands (n = 5 plots per stand) at Huitong.

Parameter	High	Low	Mixed	df	F	p-value
Basal area (m^2^ ha^-1^)						
*C*. *lanceolata*	54.20(±3.42)^a^	35.31(±2.20)^b^	23.47(±2.26)^c^	14	33.217	<0.001
*M*. *macclurei*	-	-	11.04(±1.21)			
Total	54.20^a^	35.31^b^	34.51(±2.23)^b^	14	17.219	<0.001
Stem density (trees ha^-1^)						
*C*. *lanceolata*	1130(±76.81) ^a^	1410(±101.73) ^a^	620(±86.02) ^b^	14	20.351	<0.001
*M*. *macclurei*	-	-	730(±68.19)			
Total	1130^a^	1410^a^	1350(± 47.43)^a^	14	3.524	0.063
Seedling density (stems ha^-1^)						
*C*. *lanceolata*	†	†	†			
*M*. *macclurei*	-	-	3600(±722)			
Total	-	-	3600			
Shrub density (stems ha^-1^)	5680(±833)^a^	8160(±1063)^a^	6320(±1038)^a^	14	1.713	0.222
Fern cover (total %)	9.0(±3.0)^a^	4.4(±0.9)^a^	6.4(±3.2)^a^	14	0.789	0.476
Herb cover (total %)	40.9(±12.8)^a^	27.4(±12.4)^a^	14.1(±4.1)^a^	14	1.600	0.242
Shannon’s diversity index of non-tree	1.38(±0.24)^a^	1.67(±0.22)^a^	1.67(±0.21)^a^	14	0.571	0.580

The stand types are single-species *C*. *lanceolata* with high basal area (High), single-species *C*. *lanceolata* with low basal area (Low), and mixed *C*. *lanceolata* and *M*. *macclurei* (Mixed). Different letters indicate significant differences.

†No seedling was observed.

Across stand types, Datian and Huitong showed quite different stand structure traits (Table 4). Datian had higher basal area, stem density, seedling density, and fern cover, but lower herb cover and Shannon’s diversity index of non-tree, compared with Huitong. The shrub density did not vary with the study site.

**Table 4:** Summary of the stand structure traits between sites (mean±SE).

Parameter	Datian	Huitong	df	t	p-value
Basal area (m^2^ ha^-1^)	58.09(±2.96)	41.37(±2.82)	28	4.091	<0.001*
Stem density (trees ha^-1^)	2210(±164)	1297(±53)	28	5.286	<0.001*
Seedling density (stems ha^-1^)	14167(±4013)	1200(±597)	28	3.190	0.003*
Shrub density (stems ha^-1^)	9167(±1517)	6720(±596)	28	1.501	0.145
Fern cover (total %)	77.8(±3.57)	6.6(±1.48)	28	18.425	<0.001*
Herb cover (total %)	7.9(±1.96)	27.5(±6.36)	28	-2.935	0.007*
Shannon’s diversity index of non-tree	1.24(±0.87)	1.57(±0.13)	28	-2.171	0.039*

Data was the mean value across three stand types at each site.

*Denotes significance of differences between mean values.

### Fine root biomass

At Datian, the FRBs for *C*. *lanceolata*, tree species, and total plants did not vary with stand type (Table 5). For the tree basal-area comparison, D-Low and D-High had similar total FRBs and fine-root components. Compared with the pure stand with a similar tree basal area (D-Low), the mixed stand (D-Mixed) had a lower non-tree FRB. At Huitong, stand type showed no effects on the FRBs for *C*. *lanceolata* and non-tree species (Table 5). Although the tree FRB and total FRB were higher for H-Mixed than that for H-High, the tree FRB and total FRB did not vary with the stand type for the basal-area comparison and for the comparison of single-species and mixed stands.

**Table 5:** Effects of stand type on the fine root biomass (g m^-2^) at 0–40 cm depth (mean±SE).

Species	High	Low	Mixed	df	F	p-value
Datian						
* C*. *lanceolata*	344.43(±37.84)^a^	265.42(±15.00)^a^	239.09(±39.15)^a^	14	2.827	0.099
* S*. *superba*	-	-	116.18(±18.14)			
* *Tree	344.43^a^	265.42^a^	355.27(±27.44)^a^	14	2.933	0.092
* *Non-tree	61.22(±6.83)^ab^	83.19(±11.19)^a^	48.61(±7.10)^b^	14	4.133	0.043*
* *Total	405.62(±37.66)^a^	345.78(±9.62)^a^	403.88(±34.24)^a^	14	1.145	0.351
Huitong						
* C*. *lanceolata*	57.72(±4.82)^a^	83.02(±12.49)^a^	63.12(±8.31)^a^	14	2.144	0.160
* M*. *macclurei*	-	-	37.86(±4.48)			
* *Tree	57.72^b^	83.02^ab^	100.98(±6.38)^a^	14	6.446	0.013*
* *Non-tree	24.76(±3.14)^a^	44.13(±6.28)^a^	31.68(±6.02)^a^	14	3.379	0.069
* *Total	82.48(±5.22)^b^	127.15(±18.16)^ab^	132.67(±11.91)^a^	14	4.553	0.034*

Each stand type was replicated five times. The stand types are single-species *C*. *lanceolata* with high basal area (High), single-species *C*. *lanceolata* with low basal area (Low), and mixed *C*. *lanceolata* and *S*. *superba* / *M*. *macclurei* (Mixed). Different letters indicate a significant difference.

*Denotes significance of differences between mean values.

Across stand types, Datian had higher tree, non-tree and total FRBs than Huitong (Table 6). However, the FRB of the non-tree species accounted for 16.7% and 29.4% of the total FRB at Datian and Huitong, respectively. This result indicates that the contribution of the non-tree FRB to the total FRB was higher at Huitong than at Datian.

**Table 6:** Fine root biomass (g m^-2^) at 0–40 cm soil depth between sites (mean±SE).

Species	Datian	Huitong	df	t	p-value
Tree	321.71(±18.68)	80.57(±6.59)	28	12.173	<0.001*
Non-tree	64.34(±5.98)	33.52(±3.57)	28	4.425	<0.001*
Total	385.52(±17.45)	114.10(±9.14)	28	13.780	<0.001*

Data was the mean value across three stand types at each site.

*Denotes significance of differences between mean values.

### Fine root distribution

**Fig 1:**
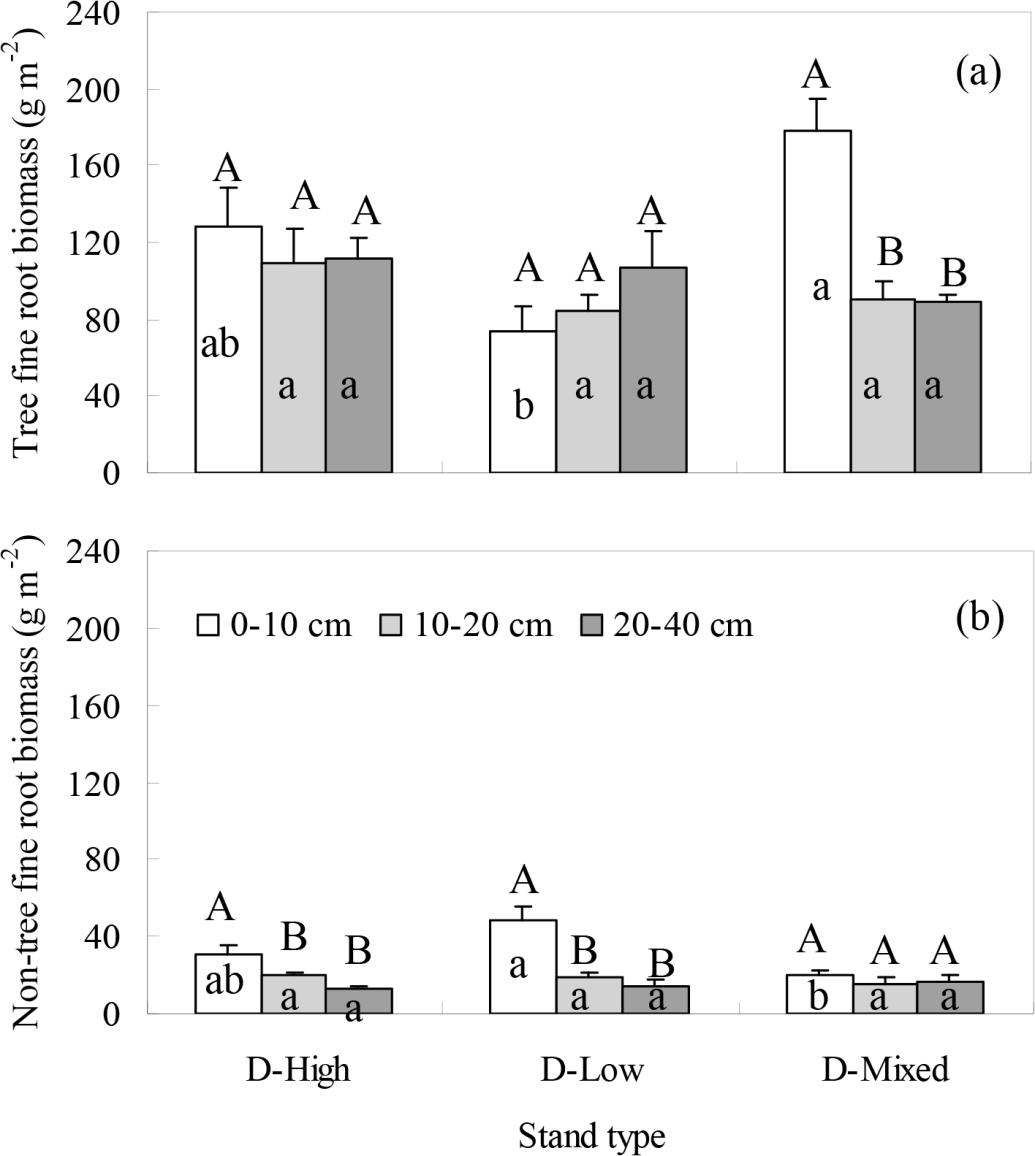
The FRBs of (a) tree and (b) non-tree species at Datian along the soil profile for five plot replicates per stand and nine sampling locations per plot. Error bar represents one standard error of the mean. Different upper-case letters denote significant differences among soil depths within stand types ((a) D-mixed: F = 20.629, P<0.001; (b) D-High: F = 9.707, P = 0.003; D-Low: F = 17.259, P<0.001). Different lower-case letters denote significant differences among stand types within soil depths ((a) 0-10cm: F = 9.108, P = 0.004; (b) 0–10 cm: 8.698, P = 0.005).

**Fig 2:**
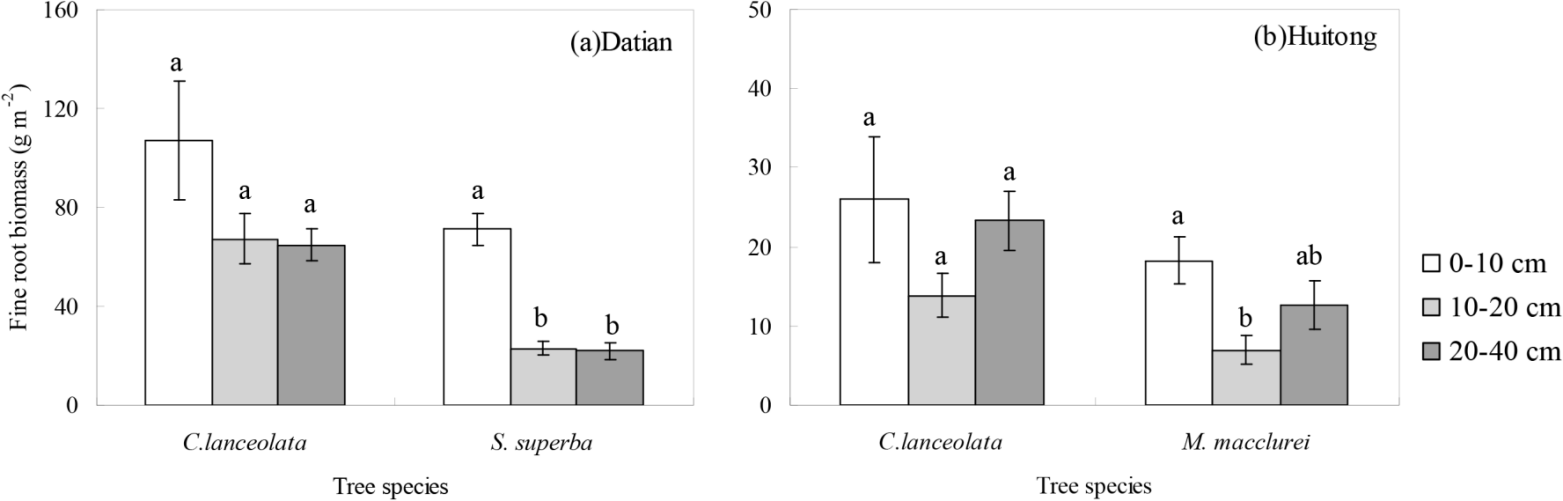
The FRBs of (a) *C*. *lanceolata* and *S*. *superba* in D-Mixed at Datian and (b) *C*. *lanceolata* and *M*. *macclurei* in H-Mixed at Huitong along the soil profile for five plot replicates per stand and nine sampling locations per plot. Error bar represents one standard error of the mean. Different letters indicate significant differences among soil depths ((a) *S*. *superba*: F = 11.260, P = 0.002; (b) *M*. *macclurei*: F = 4.536, P = 0.034).

At Datian, the distributions of tree and non-tree FRBs along the soil profile were the same between D-High and D-Low, but different between D-Low and D-Mixed (Fig 1). For D-High and D-Low, there was no significant difference in the tree FRB allocation by depth but the non-tree FRB was concentrated at 0–10 cm depth. For D-Mixed, however, the tree FRBs were more common at the upper 0–10 cm depth, and the non-tree FRB was relatively uniform along the soil profile. There was an interaction effect of soil depth and stand type on the FRB distribution of tree and non-tree species (Table 7). For D-Mixed, although the FRBs of *C*. *lanceolata* among the soil layers were not different significantly, the FRBs of *C*. *lanceolata* tended to concentrate in the upper 0–10 cm layer, as observed for *S*. *superba* (Fig 2(A)). This result indicates that a symmetrical vertical rooting pattern occurred between *C*. *lanceolata* and *S*. *superba*.

**Table 7:** Interaction effects of soil depth and stand type on the FRBs of tree and non-tree species at Datian and Huitong.

Site	Species	df	F	p-value
Datian	Tree	4	5.256	0.002*
	Non-tree	4	5.357	0.002*
Huitong	Tree	4	3.207	0.024*
	Non-tree	4	0.360	0.835

*Denotes significance of differences between mean values.

**Fig 3:**
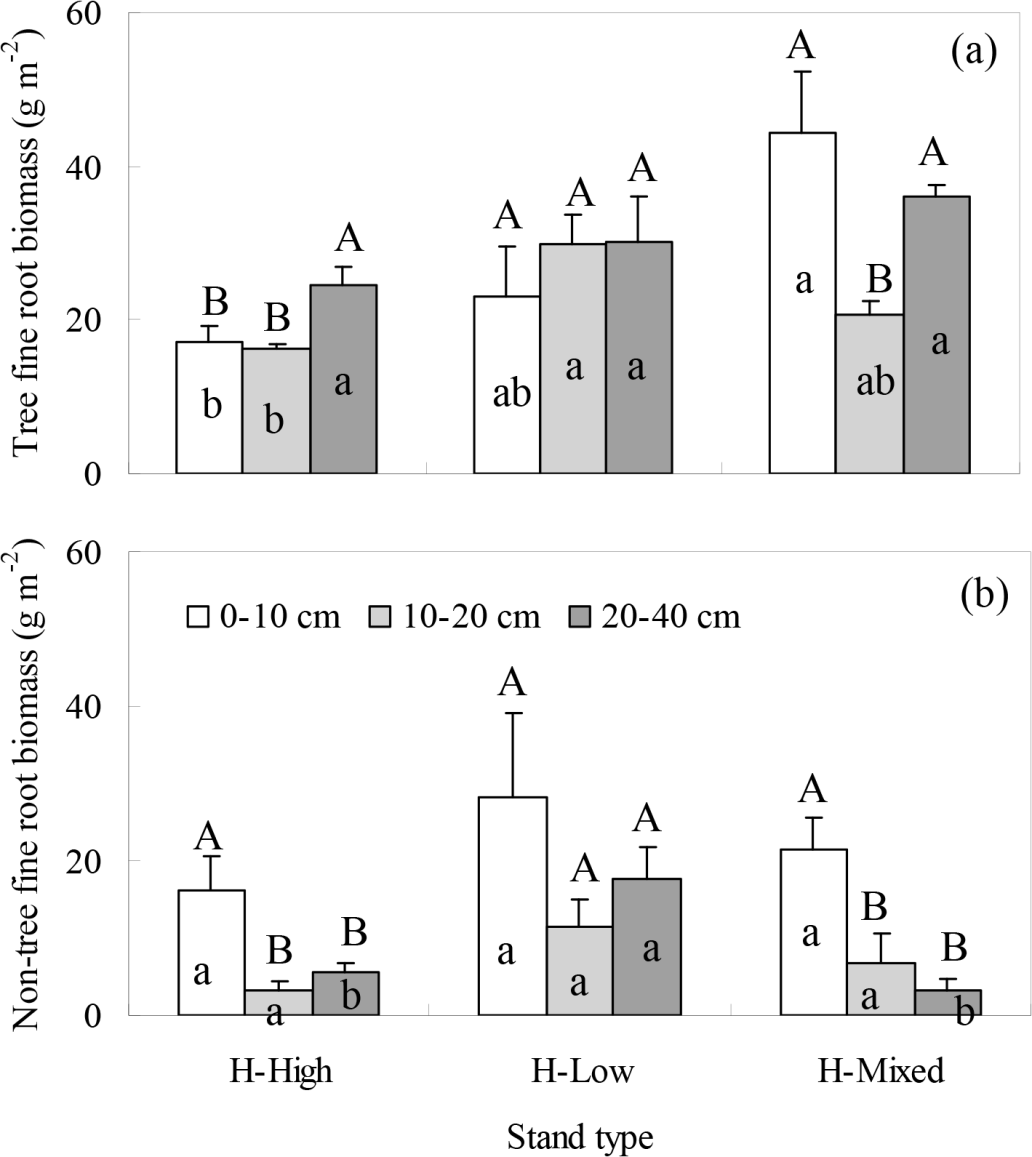
The FRBs of (a) tree and (b) non-tree species at Huitong along the soil profile for five plot replicates per stand and nine sampling locations per plot. Error bar represents one standard error of the mean. Different upper-case letters denote significant differences among soil depths within stand types ((a) H-High: F = 5.590, P = 0.019; H-Mixed: F = 5.762, P = 0.018; (b) H-High: F = 6.290, P = 0.014; H-Mixed: F = 8.795, P = 0.004). Different lower-case letters denote significant differences among stand types within soil depths ((a) 0–10 cm: F = 5.364, P = 0.022; 10–20 cm: F = 7.597, P = 0.007; (b) 20–40 cm: F = 10.004, P = 0.003).

At Huitong, the distributions of tree and non-tree FRBs along the soil profile were different among stand types (Fig 3). The tree FRB and non-tree FBR for H-High were higher in the 20–40 cm and 0–10 cm layers, respectively. The tree and non-tree FRBs for H-Low remained stable along the soil profile. For H-Mixed, however, the tree and non-tree FRBs tended to concentrate in the 0–10 cm layer. The interaction effect of soil depth and stand type on the FRB distribution was observed for the tree species but not for the non-tree species (Table 7). For H-Mixed, the FRBs of *C*. *lanceolata* and *M*. *macclurei* showed a symmetrical vertical rooting pattern (Fig 2 (B)), as observed for D-Mixed.

**Fig 4:**
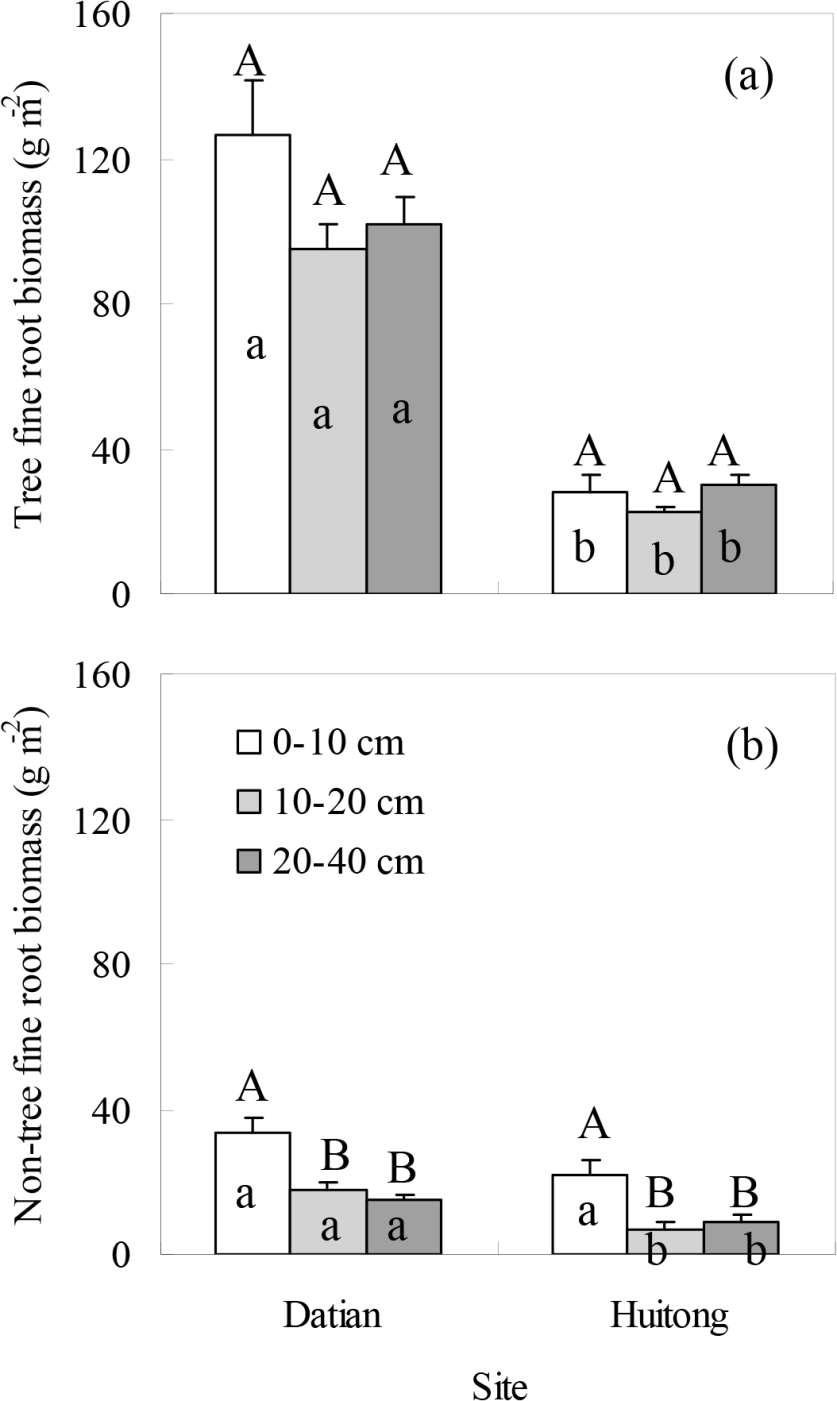
The FRBs of (a) tree and (b) non-tree species along the soil profile at the two sites. Data was the mean value across three stand types at each site. Error bar represents one standard error of the mean. Different upper-case letters denote significant differences among soil depths within sites ((b) Datian: F = 13.126, P<0.001; Huitong: F = 7.869, P = 0.001). Different lower-case letters denote significant differences between sites within soil depths ((a) 0–10 cm: t = 6.420, P<0.001; 10–20 cm: t = 9.782, P<0.001; 20–40 cm: t = 9.176, P<0.001; (b) 10–20 cm: t = 4.462, P<0.001; 20–40 cm: t = 2.265, P<0.031).

Across stand types, Datian and Huitong had similar fine root distribution along the soil profile (Fig 4). The tree FRB remained stable along the soil profile, however, the non-tree FRB concentrated in the 0–10 cm layer. Compared with Huitong, Datian had higher tree FRB along the entire soil profile but showed similar non-tree FRB at the 0–10 cm depth.

## Discussion

### Total FRB in the comparison of basal areas

Kucbel et al. (2011) reported that stands with lower basal area had lower total FRB in Norway spruce forests [34].Our results showed that the total FRB did not vary with tree basal area in the *C*. *lanceolata* plantations. This is consistent with the observations from *Quercus ilex*, pitch pine, and Japanese larch plantations [15, 16]. Vargas et al. [14], however, reported a significant increase in total FRB when basal area was removed by 17% in a dry tropical forest. The apparent contradiction in reported results on the relation between basal area and total FRB may in part reflect contrasting behavior of different tree species. Another possible explanation would be that the responses of non-tree roots to basal area depend on the thinning density since light is a crucial environmental factor affecting the density of non-tree species [32]. It logically follows that a much lower stem density would favor the growth of non-tree fine roots [16].

At Datian, D-Low had lower stem density than D-High, and the differences in stem density was around 80%. However, the cover of fern and herb for D-High was similar to that for D-Low. The total cover of fern and herb reached 89.4% and 95% for D-High and D-Low, respectively. Although the shrub density was lower for D-Low than that for D-High, the dense fern and herb resulting in no significant treatment effects on the non-tree FRBs. The similar non-tree FRBs, combined with the similar root profile between treatments, suggest that some other factors may have been responsible for the lack of variations in total FRB at this site. For example, the higher stem density for D-High may result in a severe competition between trees. In addition, the fine root biomass of a two-year old *C*. *lanceolata* seedling could be as high as 55.9 g per plant [35]. D-Low had significantly higher seedling density than D-High. The increase in seedling density for D-Low would diminish the tree FRB difference between treatments caused by the stem density difference, and thus may explain why D-Low did not experience significant reduction in the total FRB.

At Huitong, H-Low had similar stem density to H-High. The relatively low difference in stem density has shown to lead to no differences in the herb and fern cover and shrub density between treatments. Complementarity is an important factor driving the positive influence of species richness on FRB [7]. We observed that the tree FRB and non-tree FBR for H-High were concentrated at different soil layers. This result suggested that a niche complementarity occurred between the non-tree and tree species for H-High. It is likely that the quite similar stand structure between treatments may be the over-riding factor that masks the niche complementarity effect and helped in the lack of association between basal area and total FRB.

### Total FRB in the comparison of single-species and mixed stands

In contrast to previous findings that mixed forests yield more FRB than do corresponding single-species stands [2–6, 12], we found that the total FRBs at both sites were not significantly different between the mixed and single-species stands. Previous studies have observed that trees can adjust their rooting profiles asymmetrically to avoid direct competition and to maximize resource acquisition [9, 36]. The complementarity effect between tree species, however, did not appear to operate in the present study, because the mixed stands had symmetrical vertical rooting patterns between the tree species (Fig 2). We can address the symmetrical patterns in root profiles in several ways. First, although the 0–40 cm depth contained 75% of the total FRB at 0–80 cm depth [33], the root profile sampled in our study may not have been sufficiently deep to detect the niche separation between the studied tree species. Second, the study sites are fertile (S1 Table) and receive abundant rain during the study period, and root profiles can be symmetrical in environments that are not limited by water or nutrients [24, 36, 37]. This interpretation is supported by observations in multi-species broadleaved forests [11]. Third, the symmetrical pattern of the rooting profile also indicated that the fine roots of *S*. *superba* and *M*. *macclurei* have the potential to compete with those of *C*. *lanceolata*. The presence of these two broadleaved trees may not have decreased the FRB of *C*. *lanceolata* in the present study but perhaps would have if the stem densities and basal areas of the two broadleaved trees had been comparable to those of *C*. *lanceolata*. A recent study, conducted in boreal forest stands with the species of *Pinus banksiana* and *Populus tremuloides*, suggested that the evenness of tree species plays a more important role than richness in belowground productivity [4]. It needs to be studied whether or not the similarity of the total FRBs between stand types in the present study is related to the unevenness of tree species.

We believe that in addition to the lack of complementarity effect between tree species, the differences in stem density, seedling density, and belowground competition between the tree and non-tree species probably contribute to the similarity of the total FRBs in the mixed and single-species stands. At Datian, for example, D-Mixed with higher stem density did not yield higher total FRB could be due to the lower seedling density. In addition, the increase in tree FRB and the decrease of non-tree FRB at 0–10 cm depth for D-Mixed suggests that the tree species showed superiority in competition over the non-tree species at the upper soil depth in the mixed stand. At Huitong, there were no differences in the measured stand structure traits except for the seedling density. Seedling regeneration was observed for the broadleaf trees in the mixed stand but not occurred in the single species stands. The seedling roots might help to increase the FRB, to some extent, in the mixed stand. Moreover, the tree and non-tree FRBs both concentrated in the 0–10 cm layer for H-Mixed. This result suggested that a symmetrical interspecific competition between the non-tree and tree species existed in the mixed stand. The interspecific competition between the non-tree and tree species would be unfavorable for the FRB increment.

### Total FRB between sites

We found that soil fertility did not change the fine root distribution along the soil profile. Similar result was reported in northwest German old-growth beech forests [38]. The more fertile site Datian had higher tree, non-tree and total FRBs than Huitong. Clearly, the higher basal area, stem density, seedling density and total cover of fern and herb at Datian had the potential to increase the FRBs. However, studies carried out in mature forests found that infertile soil had greater FRB than nutrient rich soil to guarantee sufficient water and nutrient uptake [19, 20]. The discrepancies in the effects of soil fertility on FRB could be addressed in the following ways. First, although the soil organic mater at Huitong was lower than Datian, the basal area at Huitong was as high as 41.37 m^2^ ha^-1^, suggesting that the nutrient availability might not be a limiting factor for tree growth at Huitong. Second, the low air temperature during early growing season was the major factor controlling the carbon sequestration in subtropical coniferous forests [39]. Therefore, the lower air temperature during early growing season of the study year at Huitong likely contributed to the lower FRB. Third, an earlier study showed that the FRB was higher in pre-mature stands than in mature stands for the tree species of *C*. *lanceolata* [40]. The difference in stand stage between sites could thus help. Rather, these two sites have different soil depths and canopy closure was reported to influence the relation between site fertility and FRB [20]. The role of the differences in soil depth and canopy closure between sites in our study stands deserves further study.

Given the apparently higher total cover of fern and herb at Datian and the similar shrub density between the two sites, we expected to see a higher contribution of non-tree FRB to total FRB at Datian. However, we observed a higher contribution of non-tree FRB at Huitong. It is possible that the high level of seedling density may potentially inhibit the growth of non-tree fine roots at Datian. Our results supported the finding that the contribution of non-tree FRB to total FRB was not reflected in the visual determination of the understory cover [20].

## Conclusions

Our results have implications for the interpretation of the variation of total FRB among stand types (e.g. single-species stands with different basal areas or single-species and mixed stands with comparable basal areas). Previous studies have focused on the characteristics of the tree layer as a single mechanism that can induce variations of total FRB. In our system, these characteristics can explain a portion of the variation in total FRB, but total FRB could also be substantially influenced by the understory layer. For the basal area comparison, for example, the increase in seedling density for the low basal area stands at Datian and the quite similar stand structure between treatments at Huitong helped in the lack of association between basal area and total FRB. For the comparison of single-species and mixed stands, in addition to the lack of complementarity effect between tree species, the differences in stem and seedling densities and the belowground competition between the tree and non-tree species also contributed to the similarity of the total FRBs in the mixed and single-species stands. The results add a new context for understanding the variation of belowground yield across stand types.

## Supporting Information

S1 Table Soil chemical properties (0–40 cm depth) between sites (mean±SE). (DOC)

S1 Dataset Data underlying the findings described in the manuscript. (XLS)
