# Supplementary material for: Differences in Fine-Root Biomass of Trees and Understory Vegetation among Stand Types in Subtropical Forests
Source: PLoS One. 2015 Jun 5;10(6):e0128894. doi: 10.1371/journal.pone.0128894 (PMC4457824; doi:10.1371/journal.pone.0128894)
Supplement: S1 Table — (DOC) [file pone.0128894.s001.doc]

**S1 Table. Soil chemical properties (0-40 cm depth) between sites (mean±SE).**

| Parameter | Site | | df | t | p-value |
| --- | --- | --- | --- | --- | --- |
|  | Datian | Huitong |  |  |  |
| Soil total nitrogen (g kg^-1^) | 1.05(±0.04) | 1.05 (±0.03) | 28 | -0.052 | 0.959 |
| Soil organic carbon (g kg^-1^) | 21.86(±0.92) | 15.61(±0.53) | 28 | 5.397 | <0.001* |

Data was the mean value across three stand types at each site.

*Denotes significance of differences between mean values.
